# Comparative Analysis of Transposable Elements and the Identification of Candidate Centromeric Elements in the *Prunus* Subgenus *Cerasus* and Its Relatives

**DOI:** 10.3390/genes13040641

**Published:** 2022-04-02

**Authors:** Lei Wang, Yan Wang, Jing Zhang, Yan Feng, Qing Chen, Zhen-Shan Liu, Cong-Li Liu, Wen He, Hao Wang, Shao-Feng Yang, Yong Zhang, Ya Luo, Hao-Ru Tang, Xiao-Rong Wang

**Affiliations:** 1 College of Horticulture, Sichuan Agricultural University, Chengdu 611130, China; ewlei@163.com (L.W.); wangyanwxy@sicau.edu.cn (Y.W.); 71281@sicau.edu.cn (J.Z.); fengyan@stu.sicau.edu.cn (Y.F.); supnovel@sicau.edu.cn (Q.C.); l33yona@163.com (Z.-S.L.); hewen@sicau.edu.cn (W.H.); wh2sky@163.com (H.W.); 71175@sicau.edu.cn (S.-F.Y.); zhyong@sicau.edu.cn (Y.Z.); 13621@sicau.edu.cn (Y.L.); htang@sicau.edu.cn (H.-R.T.); 2 Institute of Pomology and Olericulture, Sichuan Agricultural University, Chengdu 611130, China; 3 Zhengzhou Fruit Research Institute, Chinese Academy of Agricultural Sciences, Zhengzhou 410100, China; liucongli@caas.cn

**Keywords:** *Prunus* (*Cerasus*), long terminal repeat retrotransposons (LTR-RTs), tandem repeats (TR), Oligo-FISH, centromere-associated repetitive sequence

## Abstract

The subgenus *Cerasus* and its relatives include many crucial economic drupe fruits and ornamental plants. Repetitive elements make up a large part of complex genomes, and some of them play an important role in gene regulation that can affect phenotypic variation. However, the variation in their genomes remains poorly understood. This work conducted a comprehensive repetitive sequence identification across the draft genomes of eight taxa of the genus *Prunus*, including four of the *Prunus* subgenus *Cerasus* (*Prunus* *pseudocerasus*, *P. avium*, *P. yedoensis* and *P. × yedoensis*) as well as congeneric species (*Prunus salicina*, *P. armeniaca*, *P. dulcis* and *P. persica*). Annotation results showed high proportions of transposable elements in their genomes, ranging from 52.28% (*P. armeniaca*) to 61.86% (*P. pseudocerasus*). The most notable differences in the contents of long terminal repeat retrotransposons (LTR-RTs) and tandem repeats (TRs) were confirmed with *de novo* identification based on the structure of each genome, which significantly contributed to their genome size variation, especially in *P. avium* and *P.* *salicina*. Sequence comparisons showed many similar LTR-RTs closely related to their phylogenetic relationships, and a highly similar monomer unit of the TR sequence was conserved among species. Additionally, the predicted centromere-associated sequence was located in centromeric regions with FISH in the 12 taxa of *Prunus*. It presented significantly different signal intensities, even within the diverse interindividual phenotypes for *Prunus tomentosa*. This study provides insight into the LTR-RT and TR variation within *Prunus* and increases our knowledge about its role in genome evolution.

## 1. Introduction

*Cerasus* belongs to the Rosaceae family, and is an important subgenus of the genus *Prunus*. This genus contains many crucial economic drupe fruits, such as peach, mei, plum, apricot and almond, which are mainly consumed when fresh around the world because of their high nutritional value and desirable taste [1]. Many species of this genus also present many high ornamental and economic value for their flowers, which are loved by people all over the world [2,3]. The genus *Prunus* consists of more than 250 species, most of which are diploid in cultivated species, except for tetraploid Chinese cherry (*P. pseudocerasus*), sour cherry (*P. cerasus*) and hexaploid European plum (*P. domestica*).

Repetitive sequences represent the predominant fraction of plant genomes, and they have been used for assessing interspecific phylogenetic relationships and evolution [4,5,6]. Their distribution is mainly divided into dispersed and tandem repeat (TR) sequences [7]. Transposable elements (TEs) constitute almost all of the repetitive DNA dispersed in plant genomes [8]. Long terminal repeat retrotransposons (LTR-RTs) account for the majority of TEs [9], and these elements show an astonishing rate of amplification and removal that drives genome size evolution [10,11] or agronomic [9,12] trait changes. TR sequences mostly accumulate in certain positions or regions [13], are essential for genome stability and play roles in centromere function, meiotic chromosome segregation, and gene regulation [14]. They are also valuable as a source of cytogenetic markers for cytological investigation in regions such as telomeres, subtelomeres, rDNAs and centromeres [15].

Many high-quality genomes have been sequenced in recent years, generating reads of up to several tens of kilobases or even 1 Mb [16], and providing superior performance in repeated sequence analysis. Over the last ten years, the genomes of many species of the genus *Prunus* have been sequenced, including those of mei [17], peach [18,19,20], sweet cherry [21,22,23], flowering cherry [2,3,24,25], almond [26,27], apricot [28], plum [29,30] and Chinese cherry (unpublished). Most of these efforts involved single-molecule sequencing combined with high-throughput sequencing, which provides high-quality genomes. Thus, it has become possible to better understand repetitive sequences, providing valuable evolutionary information about repeat sequences among species. However, disparities among the identification methods and genome qualities have been described in these genomes.

The main goal of this study was to provide an overview of repetitive sequences in the *Prunus* subgenus *Cerasus* and its relatives, and to specifically characterize the effects of LTR-RTs and TRs on genome composition. We performed a comprehensive analysis of repetitive sequences using eight genome assemblies of *Prunus*. This process combines homology- and structure-based methods, comparing their abundance and sequence similarity across species. We additionally predicted centromere-associated satellite sequences based on the characteristics of the centromeres, which were confirmed to be highly conserved, through the use of fluorescence in situ hybridization (FISH) technology, among ten species belonging to the genus *Prunus*. The variability of repetitive sequences will provide insight into genome evolution and systematic implications.

## 2. Materials and Methods

### 2.1. Genome Dataset and Plant Material

This study selected the genomes of eight taxa of the *Prunus* subgenus *Cerasus* and its relatives to analyze their repetitive sequences (Table 1). Detailed genome assembly information is shown in Supplementary Table S1. We assembled a draft genome of *P. pseudocerasus* (unpublished). The genomes of flowering cherry ‘Somei-Yoshino’ (*P. × yedoensis*, v3.1) [24], peach (*P. persica*, v2.0.a1) [19], plum (*P. salicina*, v1.0) [29], apricot (*P. armeniaca*, v1.0) [28] and almond (*P. dulcis*, v2.0) [27] were downloaded from the Genome Database for Rosaceae (GDR) database. The genomes of sweet cherry ‘Tieton’ (*P. avium*) [23] and wild flowering cherry ‘Pyn-Jeju2′ (*P. yedoensis*, v1.0) [2] were downloaded from the NCBI database (the National Center for Biotechnology Information). Additionally, sequencing reads of ten species were also used to identify satellite DNAs (Supplementary Table S2). Moreover, we collected the seeds of 13 accessions to conduct molecular cytogenetic analyses (Table 1).

### 2.2. TE Identification, Classification and Annotation

TEs were identified using de novo methods in the high-quality representative genomes of four taxa (genome quality was assessed based on sequencing technology, assembly continuity and completeness), including *P. pseudocerasus*, *P. avium*, *P. × yedoensis* and *P. persica* with EDTA (v1.8.3) [31] and RepeatModeler2 [32]. Subsequently, they were merged to generate a draft TE database, and unknown sequences were classified into superfamilies with DeepTE [33]. Among the TEs, those with low complexity, satellites, simple repeats and sequences with lengths of less than 80 nt were discarded. Highly similar sequences were removed using CD-HIT (v 4.8.1) [34] (with the following parameters: -aS 0.8 -aL 0.8 -c 0.8) to obtain a final TE library. Then, the library was used to annotate the genomes of eight taxa of *Prunus* with RepeatMasker (http://repeatmasker.org, accessed on 3 February 2020, version 4.0.7) with the settings ‘-q -no_is -norna -nolow’.

### 2.3. Full-Length Long Terminal Repeat Retrotransposon Identification

The canonical full-length LTR-RTs of the assembled genomes of eight taxa were predicted using LTR_FINDER (v1.0.7) [35], ltrharvest (v1.5.10) [36] and LtrDetector (v1.0) [37]. The parameters were set as follows: LTR length ranging from 100 to 8000 bp, the distance between LTR start positions ranging from 400 to 25,000 bp and a similarity threshold of 0.85. False-positive LTR elements were filtered out in LTR_retriever (v2.8) [38] with the default parameters. Similar LTR elements were clustered into families with CD-HIT (v4.8.1) [34] to explore their sequence diversity if they shared more than 80% sequence identity, and an alignment covering each family was generated. LTR-RTs were annotated based on the nonredundant LTR library generated from LTR_retriever by RepeatMasker with the default parameters. Divergence times were estimated by alignment based on two LTR regions of TEs following the approach described in LTR_retriever [38], and a *Populus trichocarpa* mutation rate of 7.77 × 10^−9^ per site per year was set as the substitution rate [39].

### 2.4. Tandem Repeats and Centromere Prediction

TR sequences of the assembled genomes of eight taxa were identified using the Tandem Repeats Finder (TRF) algorithm [40] with alignment parameters of 2, 7 and 7 for matches, mismatches and delta, respectively, and a minimum alignment score of 50. The types, numbers and contents of these sequences were analyzed with a Perl program (period size > 11, copy number > 10 and percent matches > 80) implemented in the Tandem Repeats Analysis Program (TRAP) [41]. CD-HIT was employed to screen high-copy-number TR sequences to cluster those with lengths between 100 bp and 600 bp according to a sequence identity of 80%.

Meanwhile, a randomly selected sequencing dataset representing an approximately 0.3× genome size was used for the identification of genomic tandem repeats with graph-based clustering using TAREAN (Tandem Repeat Analyzer, https://repeatexplorer-elixir.cerit-sc.cz/galaxy/, accessed on 15 January 2020) [42], which contains 500,000 paired-end reads without considering the differences in genome size and ploidy. Their proportion was calculated as the percentage of reads in the cluster with respect to the number of analyzed sequences.

Centromeric regions are usually enriched in tandem repeats. Moreover, the candidate centromeric repeat of peach has already been reported to have a monomer length of 166 bp [43]. Here, we focus on highly abundant TR sequences with unit lengths ranging between 100 and 350 bp to predict centromeres in the genomes of eight taxa. Homologous TR sequences were further confirmed by dot plot analysis using JDotter [44], and multiple alignments of identified monomer units were performed with ClustalX2.

### 2.5. Chromosome Preparation, Probe Synthesis and FISH

Materials processing was performed following the methods of Wang [45], with minor modifications. Chromosomes were prepared via the slide-drop method. Briefly, root tips from germinated seeds were dissected and digested in 2% cellulase and 1% pectinase (Y23, Yakult) at 37 °C for 30–50 min. Then, they were crushed and separated from the mixture. Isolated root tip pieces were dissolved in 50 µL of glacial acetic acid. The cell suspension (8–10 µL) was dropped onto glass slides and dried slowly at room temperature.

The oligonucleotide probes of an *Arabidopsis*-type telomere repetitive sequence (TAMRA-5′ TTTAGGGTTTAGGGTTTAGGG-3′) and a conserved candidate centromere repetitive sequence from *Prunus* (FAM-5′-GTAGTTCTAGCGATTGGATTTCACTCAAAACTCACCAAATGACTCCTCCCACCATATTA-3′) were synthesized by Generay (Shanghai, China).

FISH was performed according to a previously reported method, with slight modifications [46]. The hybridization mixture consisted of 50% (*v*/*v*) deionized formamide, 10% (*w*/*v*) dextran sulfate, 2 × SSC and 50 ng of each oligo probe. A 20 µL mixture was added to the chromosome slide, covered with a coverslip, denatured for 5 min at 85 °C and incubated at 37 °C for 4–6 h. Subsequently, the hybridized chromosomes were washed with 2 × SSC for 5 min at 37 °C and then for an additional 5 min at room temperature. Chromosomes were counterstained in 2 ng µL^−1^ DAPI (4′,6-diamidino-2-phenylindole) in Vectashield antifade medium (Solarbio, Beijing, China) after the slides had dried.

Photographs were taken with a DP-70 CCD camera attached to a BX53 fluorescence microscope (Olympus, Tokyo, Japan), and images were captured with Olympus CellSens Standard Software. If necessary, the images were processed to adjust their brightness and contrast using ImageJ software.

The specific probes unambiguously confirmed the sequence location on FISH analysis. Therefore, we employed the probe sequence to perform BLAST searches (-task blastn-short -word_size 7 -evalue 1) against draft genomes to visualize their distribution to determine the candidate centromeric position accuracy and precision.

### 2.6. Identification and Phylogenetic Analysis of Centromeric Histone H3

The CENH3 protein with a centromere-targeting domain (CATD) binds to centromeric sequences defining the centromeres. In order to gain further insight into the relationship between centromeric sequences and CENH3 protein, we carried out the identification and sequence analysis of CENH3 protein. Eight different types of CENH3 protein sequences in *Arabidopsis thaliana* (gi: 75180943, 27805477, 27734400, 119370650, 75172979, 75263170, 75333996 and 75158588) were downloaded from the NCBI Protein Database (http://www.ncbi.nlm.nih.gov/protein, accessed on 10 November 2020). They were aligned with the protein sequences of *P. avium*, *P. yedoensis*, *P. × yedoensis*, *P. salicina*, *P. armeniaca*, *P. dulcis* and *P. persica* to search their CENH3 protein sequences. Subsequently, the predicted CENH3 protein sequences were confirmed in the databases Pfam (http://pfam.xfam.org/, accessed on 10 November 2020), CDD (https://www.ncbi.nlm.nih.gov/cdd/, accessed on 10 November 2020), SMART (http://smart.embl.de/, accessed on 10 November 2020) and HistoneDB 2.0 (https://www.ncbi.nlm.nih.gov/research/HistoneDB2.0/, accessed on 10 November 2020). The CENH3 protein sequences from Rosales (*Armeniaca mume*, *Pyrus × bretschneideri*, *Malus domestica*, *Fragaria vesca* and *Rosa chinensis*) and *Ziziphus jujuba* were downloaded from the NCBI database. Sequence alignment was performed using the local BLAST tool to predict CDSs from the genome of *P. pseudocerasus*. Sequence alignments were performed by ClustalX2. A neighbor-joining phylogenetic tree was generated with MEGA-X, using the best-fit model and 1000 bootstrap replicates, and the CENH3 protein sequences from *Ziziphus jujuba* were used as outgroups.

## 3. Results

### 3.1. Comparative Analysis of Transposable Elements

The final TE library contained 22,990 sequences, generated according to the method described in the Materials and Methods section, from the genomes of *P. pseudocerasus*, *P. avium*, *P. × yedoensis* and *P. persica* (Supplementary Table S3). The TEs accounted for more than half of each genome in the eight assembled genomes (Table 2). Significant differences in content were observed. They presented slightly higher proportions in the *Prunus* subgenus *Cerasus*, ranging from 55.81% for *P. avium* to 61.86% for *P. pseudocerasus*. However, this figure was lower in other closely related species, ranging from 52.28% (*P. armeniaca*) to 54.41% (*P. persica*), except for *P. salicina* (58.05%). Moreover, higher TE content in the regions of pseudochromosomes corresponded to genes with areas of lower coverage, which showed a negative correlation (Supplementary Figure S1).

Across TE families (or superfamilies), most elements showed similar contents among species, except for LTR-RTs. The genomes of tetraploid *P. pseudocerasus* and two diploid species, *P. avium* and *P. salicina*, showed a higher proportion of LTR-RTs, with 27.66%, 29.03% and 31.38%, respectively. In contrast, the other genomes presented lower proportions, ranging from 21.16% (*P. armeniaca*) to 24.87% (*P. × yedoensis*_spa). This difference might be due to a great amplification of transposable elements, especially in *P. avium* and *P. salicina*. Interestingly, the content of PIF-Harbinger was originally annotated as abnormal in *P. avium* (18.21%); for other genomes, the content was significantly lower, ranging from 3.12% (*P. salicina*) to 4.15% (*P. armeniaca*), except for *P. × yedoensis* (4.92% and 8.52%, respectively). Subsequently, we manually inspected the annotation results. A sequence was abnormally annotated, accounting for 12.65% in *P. avium*, while it was 1% in other genomes, except for *P. × yedoensis*_spa (3.38%). Finally, this repeat sequence has been demonstrated to contain a partial tandem repeat with a conserved monomer unit of 166 bp. This may be due to the complexity of the structure of the repeat sequence. After removing it, the content of PIF-Harbinger was similar among genomes.

### 3.2. Characterization and Similarity of LTR-RT Sequences

**Figure 1:**
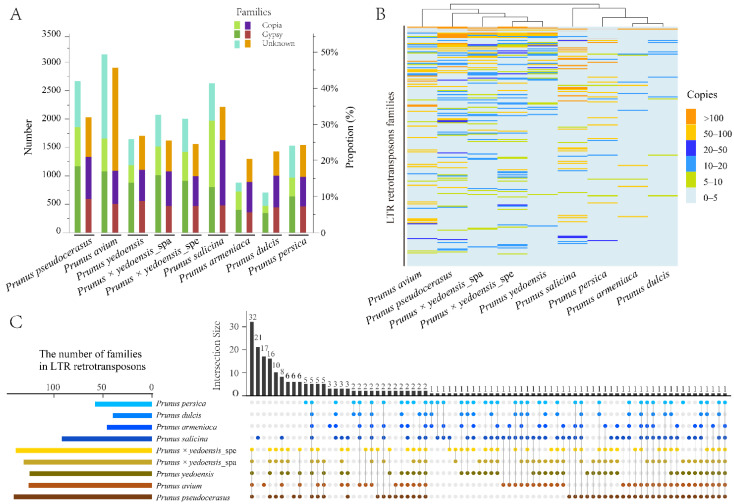
Analyses of LTR-RTs in nine haploid genomes. (**A**) The copy numbers (N) of full-length LTR-RTs and proportions (P) of TLR-RT content in genomes. (**B**) Copy number differences of full-length LTR-RTs in different clusters among genomes. (**C**) UpSetR of the LTR-RT clusters shared by the *Prunus* subgenus *Cerasus* and its relatives. Intersection sizes on the vertical bars represent the numbers of LTR-RT clusters for a given pattern. The horizontal bars on the left show the whole clusters of LTR-RTs detected in each species. Datasets appearing in intersections are shown with spots.

We comprehensively identified 17,273 copies of full-length LTR-RTs from eight taxa genomes, and their copy numbers and contents exhibited significant variation among species (Figure 1A, Supplementary Table S4). Relatively high copy numbers and contents were observed in tetraploid *P. pseudocerasus* (2665/31.95%), diploid *P. avium* (3140/45.61%) and *P. salicina* (2629/34.72%), while much lower copy numbers and contents were observed in *P. armeniaca*, *P. dulcis* and *P. persica* (copy number: 706–1529, content: 20.37–24.20%). Similar LTR-RT contents (~25%) were detected in the *P. yedoensis* and *P. × yedoensis* flowering cherries. Moreover, the distance from the inserted full-length LTR-RTs to the nearest gene across the species was mainly concentrated within 5 kb, and the content was similar in the upstream and downstream regions of the gene (Supplementary Figure S2).

All full-length LTR-RTs (17,273) were clustered into 7279 families based on sequence similarities (Supplementary Table S5). Only 1118 (15.36%) families were species-specific, while two or more species shared the others. After the families with more than ten copies among species were screened for a detailed comparison, 5694 copies were clustered into 230 families, 176 (76.52%) families were shared among the species and two families were present in all of the species (Figure 1B,C, Supplementary Table S6). The number of shared LTR-RTs of most families among the species was highly variable, particularly in diploid *P. avium* and *P. armeniaca*, which have one species-specific cluster containing more than one hundred copies. However, four *Prunus* subgenus *Cerasus* species (*P. pseudocerasus*, *P. avium*, *P. yedoensis* and *P. × yedoensis*) that shared many LTR-RT families were clustered into a single clade. Similar findings were obtained from their relatives (*P. salicina*, *P. armeniaca*, *P. dulcis* and *P. persica*), implying that LTR-RT sequence similarity is closely related to phylogenetic relationships.

### 3.3. LTR Insertion Time Estimation

**Figure 2:**
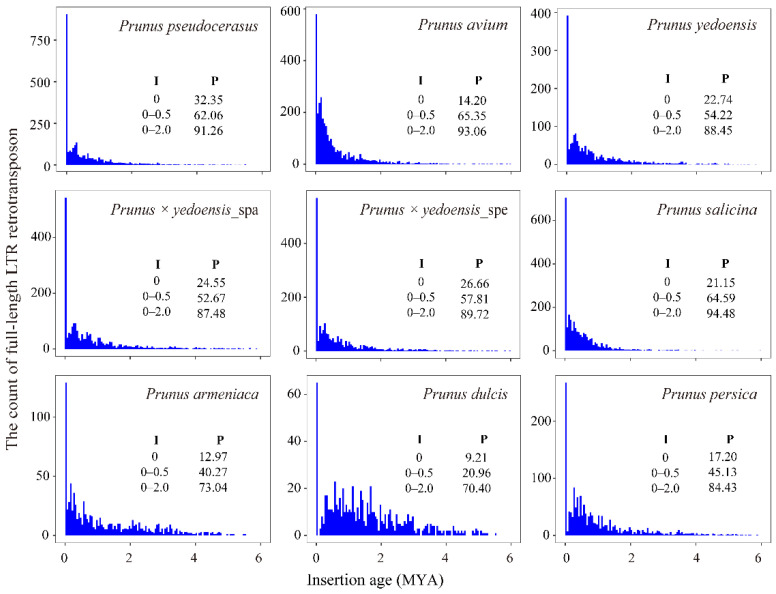
Distribution of the insertion ages of LTR-RTs. I represents insertion age; P represents the percentage (%). Insertion time was split into bins of 0.05 MYA.

The insertion ages of LTR-RTs were estimated according to nucleotide substitution to gain insight into their evolution. It can be noted that the majority of LTR-RTs (from a minimum of 70.40% in *P. dulcis* to a maximum of 94.48% in *P. salicina*) were inserted approximately 2 million years ago (MYA) (Figure 2), and they occurred after species divergence. In general, similar insertion time patterns were observed among these eight species. However, it should be noted that a massive amplification of LTR-RTs is currently underway (0.0 MYA), suggesting that different levels of activity are maintained among LTR-RTs. In particular, for tetraploid *P. pseudocerasus* and two interspecific diploid hybrid flowering cherry species, *P. yedoensis* and *P. × yedoensis*, the ratio of recently inserted elements was more than 20%, while it was less than 20% in other genomes, except for *P. salicina* (21.15%).

### 3.4. Characterization of Tandem Repeats

**Figure 3:**
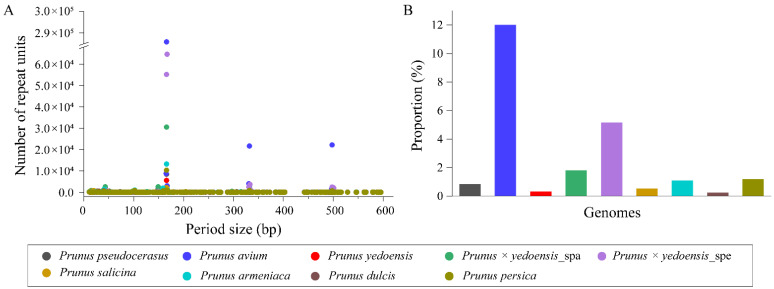
Analyses of TR sequences in nine haploid genomes of eight taxa. (**A**) Length distribution of TRs in the genomes; (**B**) TR contents in the genomes.

The TR sequence length distribution was similar among different genomes and was mainly concentrated at 165–167 bp, 331–334 bp and 497–501 bp (Figure 3A). Sequence cluster analyses showed that most of them contained a conserved monomer unit of 166 bp. However, their contents varied widely among different genomes, with the highest proportion being observed in *P. avium* (12.00%) and being much lower in the remaining species, ranging from 0.23% (*P. dulcis*) to 1.18% (*P. persica*), except for *P. × yedoensis* (1.79% and 5.17%, respectively) (Figure 3B).

Further analyses with unassembled sequences of these species were also used to determine the tandem repeats by TAREAN (Supplementary Table S7). A high content of the monomer sequence was identified to be 166 bp in *P. avium* (15%) and *P. armeniaca* (6.9%), and it was lower than 4% in the remaining species. Among these, its content between the intraspecies of two different phenotypes of *P. tomentosa* was comparatively distinct (0.23% in the white fruit and 2.3% in the red fruit). Subsequently, dot plot comparison of the monomers of 166 bp was performed in different species, revealing that they were highly conserved (Supplementary Table S8, Supplementary Figure S3).

Overall, a highly conserved monomer unit sequence of 166 bp was identified from both the assembled genomes and sequencing reads. Coincidentally, this unit was similar to the candidate centromeric repeat identified previously in peach [43]. If these sequences are confirmed to be the centromeric repeats of these species, our results would indicate that these eight taxa exhibit similar centromere-associated repeat sequences.

### 3.5. Centromeric and Telomeric Distribution on Chromosomes

**Figure 4:**
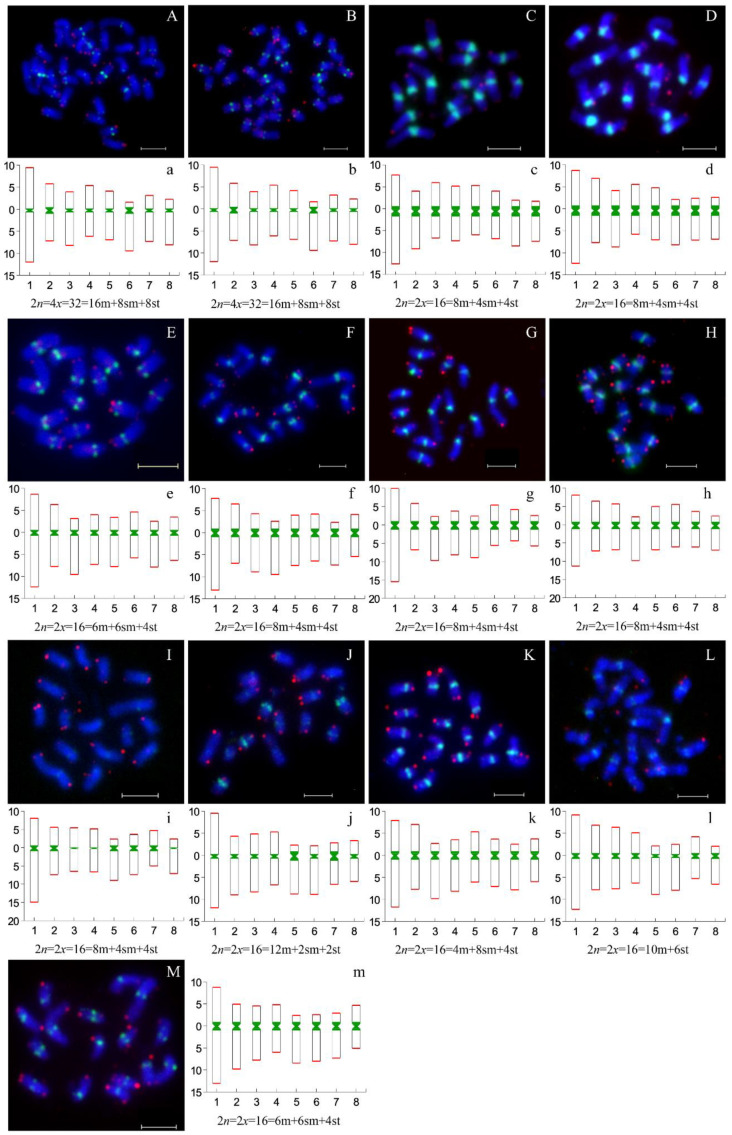
Chromosomal distribution of centromeres and telomeres among *P. pseudocerasus* and nine relatives. Green and red signals represent the distributions of centromeres and telomeres, respectively. (**A**–**M**) Chromosomal distribution of centromeres and telomeres of different species. a–m: Ideigram and karyotype formula of different species. (**A**(**a**),**B**(**b**)) *P. pseudocerasus*, HC and XC1; (**C**(**c**),**D**(**d**)) *P. avium*, ‘Mazzard’ and ‘Van’; (**E**(**e**)) *P. campanulate*, Pcampan; (**F**(**f**)) *P. yedoensis*, Pyedoensis; (**G**(**g**)) *P. humilis*, Phumilis; (**H**(**h**),**I**(**i**)) *P. tomentosa*, red and white fruit; (**J**(**j**)) *P. salicina*, ‘Cuihongli’; (**K**(**k**)) *P. armeniaca*, ‘diaogan’; (**L**(**l**)) *P. dulcis*, Pdulcis; and (**M**(**m**)) *P. persica*, Ppersica.

The FISH technique was used to directly detect the distribution patterns of the monomer unit of 166 bp and the telomeric repetitive sequences on metaphase chromosomes of thirteen accessions of the *Prunus* subgenus *Cerasus* and its relatives. The telomeric signals were mainly located at the ends of chromosomes (Figure 4, Supplementary Figure S4), but weaker (or even absent) signals were occasionally detected on some chromosomes. The monomer unit of 166 bp exhibited different signal intensities in the centromeres of chromosomes for most species. This finding implies that centromeric regions are enriched in this repeated sequence. Furthermore, the number of copies of this repetitive sequence varies between species or even between chromosomes within species (Figure 4). The most vital signals of the centromere probe were detected in the centromeric regions on cultivated and wild species of *P. avium* (Figure 4C,D). Meanwhile, we observed that the signals of the centromeric probe were variable between the two taxa of *P. tomentosa*; that is, the red fruit type exhibited strong signals at the centromeres (Figure 4H), while the white fruit type exhibited weaker signals or even a complete lack of signals on some chromosomes (Figure 4I). This result is consistent with the content of satellite DNA that was detected by TAREAN using sequencing reads. Additionally, *P. pseudocerasus*, *P. salicina* and *P. dulcis* displayed extremely weak signals on certain chromosomes. Among the remaining materials, a clear signal was observed at the position of centromeres.

**Figure 5:**
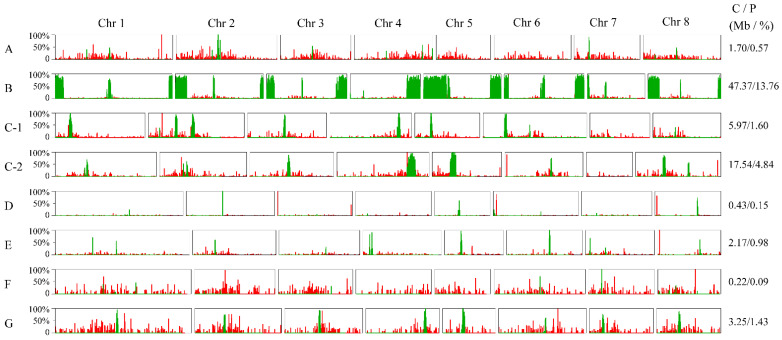
Distribution of centromere-associated and telomeric repetitive sequences on pseudochromosomes. Green and red bars represent the centromere and telomere sequences, respectively. The Y-axis indicates the percentage. (bin width: 100 k, step size: 50 k). (**A**) *P. pseudocerasus*; (**B**) *P. avium*; (**C-1**) *P.* × *yedoensis*_spa; (**C-2**) *P.* × *yedoensis*_spe; (**D**) *P. salicina*; (**E**) *P. armeniaca*; (**F**) *P. dulcis*; and (**G**) *P. persica*. The contents and proportions of the centromere sequences are shown on the right side for each haploid genome. C represents contents (Mb); P represents percentages (%).

The sequences of centromeric and telomeric regions could be intuitively seen on their pseudochromosomes through sequence alignment (Figure 5 and Supplementary Table S9). Telomeric sequences were mainly dispersed across the pseudochromosomes rather than concentrated in terminal regions, as shown in the FISH results, and their contents were relatively low in the assembled genomes, ranging from 0.0050% (*P. persica*) to 0.0218% (*P. pseudocerasus*). In parallel, the candidate centromere sequences were enriched on the pseudochromosomes in *P. persica*, with a location which is consistent with the FISH result, indicating that a relatively complete candidate centromere was assembled in this genome (Figure 4 and Figure 5). Notably, the highest content of centromere sequences was detected in *P. avium* (assembled genome: 13.76%, sequencing read: 15%). An unexpected observation was that most locations of the 166 bp monomer were apparently incorrectly assembled, according to FISH signals. According to the FISH results and satellite DNA identification from the sequencing reads, in some pseudochromosomes in the remaining species the 166 bp sequences were underestimated or the sequences were misplaced in the assembly. For example, the content of the 166 bp repetitive DNA was much lower in the assembled genomes of *P. dulcis* (0.09%) and *P. armeniaca* (0.98%), while it was higher in their sequencing reads (3.10% in *P. dulcis*, 6.90% in *P. armeniaca*); additionally, their FISH signals were more intense than in other species with a higher amount of these sequences.

### 3.6. Conservation of Centromere-Specific Histone H3

**Figure 6:**
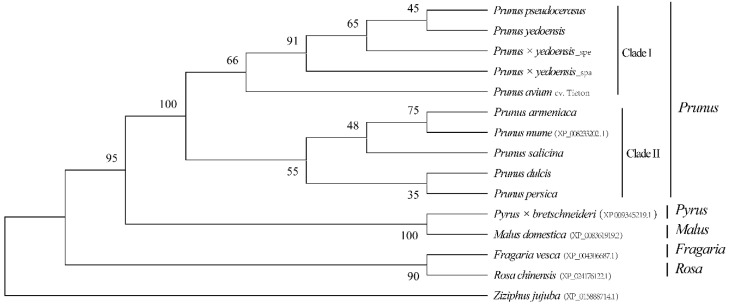
Phylogenetic tree based on the amino acid sequences of CENH3. NJ phylogenetic trees were reconstructed using MEGAX with the Jones–Taylor–Thornton (JTT) evolutionary model. CENH3 from *Ziziphus jujuba* was used as an outgroup. The numbers on the nodes correspond to bootstrap values based on 1000 tests. Only values higher than 30% are shown.

CENH3 with 172 amino acids was identified among the eight draft genomes. The alignment was conserved at the histone fold domain compared to other plant species of Rosaceae, not at the N-terminal domain (Supplementary Figure S5), hinting that they have expanded from a common ancestor. As expected, the phylogenetic analysis of CENH3 showed that the four *Cerasus* taxa (*P. pseudocerasus*, *P. avium*, *P. yedoensis* and *P. × yedoensis*) are closely related and were grouped together, and a similar result in the five taxa, including *P. mume*, *P. armeniaca*, *P. salicina*, *P. dulcis* and *P. persica*, formed another clade, constituting the sister clade of *Cerasus* (Figure 6). The CENH3 protein from the *Pyrus*, *Malus*, *Fragaria* and *Rosa* genera compared to that of *Prunus* showed a somewhat distant phylogenetic relationship.

## 4. Discussion

### 4.1. Genome Size Expansion through the Amplification of Repetitive Sequences

Repetitive sequences are an essential component of genomes, significantly contributing to genome size variation in higher plants [47]. LTR-RTs amplified via a ‘copy and paste’ model influence and drive genome structural evolution [48]. TRs usually accumulate in centromeres, pericentromeres and telomeres, which are essential for meiotic chromosome segregation and stability [49]. As long-read sequencing technologies are applied, they can be assembled into a much more complete and contiguous genome [16]. In this study, we selected eight representative taxa genomes with completeness and quality for repetitive sequence comparison (Supplementary Table S1), which showed more significant variations in LTR-RTs and TRs among species. In particular, the repetitive sequences displayed a similar pattern of distribution on their pseudochromosomes across different species, and they exhibited a higher proportion in regions with lower gene density. These results suggested that the location of repetitive sequence expansion did not appear randomly. Large genome sizes with high contents of repetitive sequences have been found in *P. avium* (the estimated genome size was 340 Mb, the LTR accounted for 45.61% and the TR accounted for 12.00%) and *P. salicina* (the estimated genome size was 312 Mb, the LTR accounted for 34.72% and the TR accounted for 0.51%). Conversely small genome sizes with low contents of repetitive sequence have been found in *P. armeniaca* (the estimated genome size was 220 Mb, the LTR accounted for 20.37%, the TR accounted for 1.09%), *P. dulcis* (the estimated genome size was 238 Mb, the LTR accounted for 22.38% and the TR accounted for 0.23%) and *P. persica* (the estimated genome size was 237–243 Mb recently, the LTR accounted for 24.20% and the TR accounted for 1.18%). The apparent correlation between repetitive sequences and genome size suggests that repetitive sequences contribute to genome size expansion.

Nevertheless, the repetitive sequence content is not positively correlated with genome size in polyploids and interspecific hybrids [50]. This was mainly due to genomic instability and genome rearrangements resulting in fragment losses or gains in chromosomes during the speciation process [51,52]. The tetraploid Chinese cherry genome size was estimated to be 294 Mb, and it was assembled to be 300 Mb. According to its genome size, the repetitive sequence content of LTR-RTs and TRs should be 10% higher than the current result (LTR accounted for 31.95%, TR accounted for 2.51%). The interspecific hybrid flowering cherry cultivars ‘Pyn-Jeju2’ and ‘Somei-Yoshino’ also showed lower proportions of repetitive sequences than the other species under study [2,24]. Peculiarly, the two haplotype-assembled genomes of ‘Somei-Yoshino’ had similar LTR accounts (25.51% and 24.48%, respectively), but were not in TR (1.79% and 5.17%, respectively).

### 4.2. LTR-RTs Drive Genome Evolution

LTR-RT elements are the primary class of repetitive sequences, and the identification of these elements will aid in the investigation of the diversity and phylogenetic evolution of TEs in plant species [53]. A comparison between the closely related species of almond and peach showed a significant difference [27]. Here, we have de novo identified full-length LTR-RTs from eight taxa genomes of the genus *Prunus*. The copy number and contents of LTR-RTs varied greatly in their genomes, being particularly high in the tetraploid *P. pseudocerasus* (2665/31.95%), diploid *P. avium* cv. Tieton (3140/45.61%) and *P. salicina* (2629/34.72%). This is mainly due to several species-specific LTR-RT amplifications that are presented according to the sequence comparison (Supplementary Tables S4 and S5). The expansion of LTR-RTs has occurred after the species divergence. The latest research suggested that the divergence times among flowering cherry, sweet cherry, Chinese cherry, almond and peach were more than five MYA ago [20,24,28]. LTR-RT bursts were detected mainly during the last two million years in this study and were consistent with those previously reported in almond, peach, apple and strawberry [27,54,55]. Among them, a higher number of new LTR-RTs were amplified (0 MYA), and we speculate that this may be due to the appearance of human domestication and selection or drastic changes in the environment, all of which would increase the activity of TEs. This study comprehensively compared the similarity of full-length LTR-RTs and revealed divergence among the genomes of *Prunus* after their speciation. Similar to many other plant species, *Gossypium* [56] and *Capsicum* [57] showed that the LTR-RT content could differ drastically even in closely related species, but the sequence similarity presented was associated with the genetic relationships of the species. Furthermore, the full-length LTR-RTs inserted into the genome were mainly concentrated within 5 kb upstream or downstream of the nearest gene. It could impact gene expression or function, as it has been recently reported that an LTR-RT insertion upstream of MdMYB1 leads to a red-skinned phenotype in apples [9]. Therefore, LTR-RTs are a potentially important source of genetic variability in the genome and may play a critical role after speciation.

### 4.3. Conservation of the Centromere Sequence and CENH3

In eukaryotes, centromeres are critical for ensuring that sister chromatids are correctly segregated during cell division [58]. They usually contain many repetitive sequences composed of satellite repeats and/or retrotransposon sequences [59]. Biased distribution of centromere-associated repetitive sequences on different chromosomes has been documented in many plants, such as radish [60], common bean [61], roses [62,63] and switchgrass [64]. Here, we found a centromere-associated repetitive domain composed of a 166 bp monomeric sequence, consistent with previous reports indicating ranges of 150 to 180 bp [43]. The centromere sequence was highly conserved among four subgenera, *Cerasus*, *Prunus*, *Armeniaca* and *Amygdalus*, without sequence similarity to rose [62,63] or black raspberry [65], even though in the closest relative genus of *Malus* [9] no significantly enriched tandem centromeric repeat was found, implying that the rapid evolution of centromere sequences was in different genera. This result is consistent with previous reports indicating limited centromere sequence similarity among species with divergence times above 50 million years [43].

However, the remarkable variation in centromere content among *Prunus* species was confirmed by satellite DNA identification with sequencing reads and FISH, and an unusual signal intensity was observed in *P. avium* (Figure 4C,D). This result illustrated that centromeres have undergone rapid independent evolution by increasing/decreasing their copy numbers after species divergence. Additionally, intraspecies analysis of two different phenotypes of *P. tomentosa* showed a clear differential pattern of signal intensity (Figure 4H,I), which was also confirmed with unassembled sequences by TAREAN (centromere sequence with 0.23% in the white fruit and 2.3% in the red fruit). The difference might be due to either centromeric DNA expansion/contraction or Robertsonian fusion/fission [66]. A similar result also emerged in wild common bean accessions [61]. However, the centromeric sequence’s function, origin and evolution are still largely unknown. The amino acid sequences of CENH3 comparison showed high conservation among the analyzed genomes, with no obvious relationship between CENH3 protein and centromere sequences. At the same time, their phylogenetic analyses were consistent with previous documents [67,68,69]. Phylogenetic relationships can be well-resolved at various taxonomic levels, as reported before in sunflower [70], cowpea [71], Secale [72] and Cyperaceae [73].

### 4.4. FISH Is a Method for Directly Correcting Assembled Genomes

FISH has become an essential method in cytogenetics because it can be used to directly visualize target sequences associated with chromosomes. It is a critical application for the localization of DNA probes on chromosomes [15]. FISH has contributed to several reports of misassembled genomes in cucumber [74,75], barley [76], tomato [77] and sacred lotus [78]. Here, the clear signals of the centromere and telomere probes observed at the metaphase chromosomes were inconsistent or underestimated with the assembled genomes (Figure 4 and Figure 5), particularly for *P. avium*, with its erroneous centromere location in the pseudochromosomes. Meanwhile, the content of the centromere-associated repetitive sequence was visibly underestimated in the remaining species, except for *P. persica*. Centromeric satellites can enrich continuously up to several Mb [58], as has been found in the present study, reaching lengths of up to 14.12 Mb on the assembled genomes of Chr_5 for *P. avium*. The centromere coverage ratio is so large that it is difficult to achieve the accurate assembly of the genome even with the recent long-read sequencing techniques and assembly methodologies available. It also indicates that the assembly completeness was why there was poor quality in *P. avium* cv Satonishiki and *P. avium* cv Big Star [21,22]. Additionally, the FISH signal and distribution of telomeres in pseudochromosomes do not corroborate. On the one hand, this may be due to incomplete telomere assembly due to its highly complex repeat arrays at the chromosome ends, as has been reported in apples [9]; on the other hand, the telomeres dispersed along the entire pseudochromosomes may be caused by sequence alignment for the short query sequence in which a large number of sites were detected. However, no telomeric signals were detected in part of the chromosome end mainly because their content was too low to be detected or the chromosome rearrangements lead to the loss of telomeric DNA during species evolution. Even though multiple orthogonal sequencing technologies were used to obtain a complete gapless chromosome (such as telomere-to-telomere genome assembly) [79], it is still challenging to obtain a higher quality genome assembly that is widespread for most species in a short time. FISH is still an efficient approach for detecting errors in assembled genomes, which will improve the quality of genome assembly for future studies.

## 5. Conclusions

Repetitive sequences are the main component discovered in the plant genome that can cause phenotypic variation. However, a more detailed study of it had not been reported in subgenus *Cerasus*. In this study, a comparative analysis of repetitive sequences in eight taxa genomes of the *Prunus* subgenus *Cerasus* and its relatives (*P. pseudocerasus*, *P. avium*, *P. yedoensis*, *P. × yedoensis*, *P. salicina*, *P. armeniaca*, *P. dulcis* and *P. persica*) improved the knowledge of their genome organization. The results showed that the contents of LTR-RTs and TRs varied remarkably and were evidently associated with genome size expansion, especially in *P. avium* and *P. salicina*. Sequence comparisons showed that many shared LTR-RTs and a conserved centromere tandem repeat sequence were found among the genomes. Additionally, the expansion of LTR-RTs mainly occurred during the last two million years. The centromere-associated sequence was confirmed with FISH in the 12 *Prunus* materials, showing that a high centromeric content was abundant in *P. avium*. The LTR-RT and TR expansions after species divergence have provided new insight into repeat sequence variation during the evolution of *Prunus*.

## Figures and Tables

**Table 1:** Reference genomes and sample information used for comparative genomics and molecular cytogenetics in this study.

Taxa	Reference Genomes	Origin and Chromosome Number of Samples Used in Molecular Cytogenetic Analyses		
		Code	Origin	Ploidy Level
subg. *Cerasus*				
*Prunus pseudocerasus*	‘Luoyang Guying’ (unpublished)	HC	Miyi, Sichuan, China	2*n* = 4*x* = 32
		XC1	Xichang, Sichuan, China	
*Prunus avium*	‘Tieton’ (v2.0) [23]	Van	ZFI, CAAS, China	2*n* = 2*x* = 16
		Mazzard		
*Prunus yedoensis*	‘Pyn-Jeju2′ (v1.0) [2]	Pyedoensis	Chengdu, Sichuan, China	2*n* = 2*x* = 16
*Prunus* × *yedoensis*	‘Somei-yoshino’ ^a^ (v3.1) [24]	-	-	2*n* = 2*x* = 16
*Prunus campanulata*	-	Pcampan	Chengdu, Sichuan, China	2*n* = 2*x* = 16
subg. *Prunus*				
*Prunus tomentosa*	-	red_fruit	ZFI, CAAS, China	2*n* = 2*x* = 16
		white_fruit		2*n* = 2*x* = 16
*Prunus humilis*	-	Phumilis	Suqian, Jiangsu, China	2*n* = 2*x* = 16
*Prunus salicina*	‘Sanyueli’ (v1.0) [29]	Cuihongli	Chengdu, Sichuan, China	2*n* = 2*x* = 16
subg. *Armeniaca*				
*Prunus armeniaca*	‘Chuanzhihong’ (v1.0) [28]	Diaogan	Akesu, Xinjiang, China	2*n* = 2*x* = 16
subg. *Amygdalus*				
*Prunus* *dulcis*	‘Texas’ (v2.0) [27]	Pdulcis	Luntai, Xinjiang, China	2*n* = 2*x* = 16
*Prunus persica*	‘Lovell’ (v2.0) [19]	Ppersica	Chengdu, Sichuan, China	2*n* = 2*x* = 16

Note: ZFI, CAAS: Zhengzhou Fruit Research Institute, Chinese Academy of Agricultural Sciences. ^a^: Two haplotype-phased genome sequences were assembled and named after CYEspachiana_r3.0 (*Cerasus × yedoensis*_spa) and CYEspeciosa_r3.0 (*Cerasus × yedoensis*_spe).

**Table 2:** Proportions of TEs and TRs in eleven haploid genomes of eight taxa in the *Prunus* subgenus *Cerasus* and its relatives.

Genome	*Prunus pseudocerasus*	*Prunus avium*	*Prunus yedoensis*	*Cerasus × yedoensis* (spa/spe)	*Prunus salicina*	*Prunus armeniaca*	*Prunus dulcis*	*Prunus persica*
LINE	1.87	1.56	1.88	1.75/1.81	1.69	1.71	1.51	1.5
SINE	0.3	0.23	0.31	0.3/0.3	0.29	0.33	0.3	0.29
LTR	27.66	29.03	24.01	24.87/23.79	31.38	21.16	24.83	23.23
Copia	10.76	8.19	9.5	9.73/9.11	9.32	7.48	9.06	9.43
Gypsy	16.9	20.84	14.51	15.14/14.68	22.06	13.68	15.77	13.8
nLTR	0.21	0.17	0.2	0.23/0.23	0.19	0.24	0.16	0.16
DIRS	0.03	0.03	0.03	0.03/0.03	0.03	0.03	0.02	0.02
PLE	0.18	0.14	0.17	0.2/0.2	0.16	0.21	0.14	0.14
Subclass_1	27.82	22.06	25.76	26.93/27.91	20.77	24.97	22.94	25.61
TIR/CACTA	8.12	4.5	6.58	6.58/7.03	4.25	4.43	5.07	7.41
TIR/MuDR	7.9	5.68	7.83	7.93/7.78	6.1	8.14	7.16	7.56
TIR/PIF-Harbinger	3.25	5.56	3.24	3.95/5.14	3.06	3.73	3.65	3.47
TIR/Tc1-Mariner	2.73	2.12	2.69	2.77/2.6	2.37	2.82	2.3	2.38
TIR/hAT	5.82	4.2	5.42	5.7/5.36	4.99	5.85	4.76	4.79
Subclass_2	4.01	2.77	3.98	3.95/3.93	3.71	3.85	3.56	3.63
Helitron	3.37	2.26	3.31	3.3/3.3	3.12	3.22	2.95	3.04
MITE	0.64	0.51	0.67	0.65/0.63	0.59	0.63	0.61	0.59
Total	61.86	55.81	56.14	58.03/57.94	58.05	52.28	53.31	54.41

## Data Availability

Not applicable.

## Supplementary Materials

The following are available online at https://www.mdpi.com/article/10.3390/genes13040641/s1, Figure S1: Distribution of genes (track with green bar), TEs (track with blue bar) and LTRs (track with brown bar) on the pseudochromosomes of Chinese cherry and related species. Figure S2: Distribution of the distance between full-length LTR-RTs and genes across the species. Figure S3: Dot plots of the identified satellite repeat sequences with monomer units of 166 bp. Figure S4: Chromosomal distribution and concentration comparison of oligonucleotide dye in 13 accessions from ten *Prunus* subgenus *Cerasus* species and related taxa. Figure S5: Alignment of the protein sequences of CENH3 from eight *Prunus* subgenus *Cerasus* and relatives. Table S1: Overview of the assembly quality and characteristics of the genomes from the *Prunus* subgenus *Cerasus* and its relatives. Table S2: The sequence reads of materials from high-throughput sequencing were used for satellite DNA identification. Table S3: Summary of TE libraries constructed with the haploid genomes of the *Prunus* subgenus *Cerasus*. Table S4: Statistics of full-length LTR-RTs in the nine haploid genomes of the *Prunus* subgenus *Cerasus* and its relatives. Table S5: The number of total full-length LTR-RT clusters among different taxa. Table S6: The number of full-length LTR-RT clusters containing more than ten copies within families. Table S7: Summary of the satellite DNAs identified by TAREAN. Table S8: The 166 bp monomer units identified in this study. Table S9: The content and proportion of all tandem repeats, centromere-associated repeats and telomeres in nine haploid genomes of eight taxa from the *Prunus* subgenus *Cerasus* and its relatives.
