# Supplementary material for: Comparative Analysis of Transposable Elements and the Identification of Candidate Centromeric Elements in the Prunus Subgenus Cerasus and Its Relatives
Source: Genes (Basel). 2022 Apr 2;13(4):641. doi: 10.3390/genes13040641 (PMC9028240; doi:10.3390/genes13040641)
Supplement: Supplementary file 1 [file genes-13-00641-s001.zip › genes-1588882-supplementary/Supplementary figures.pdf]

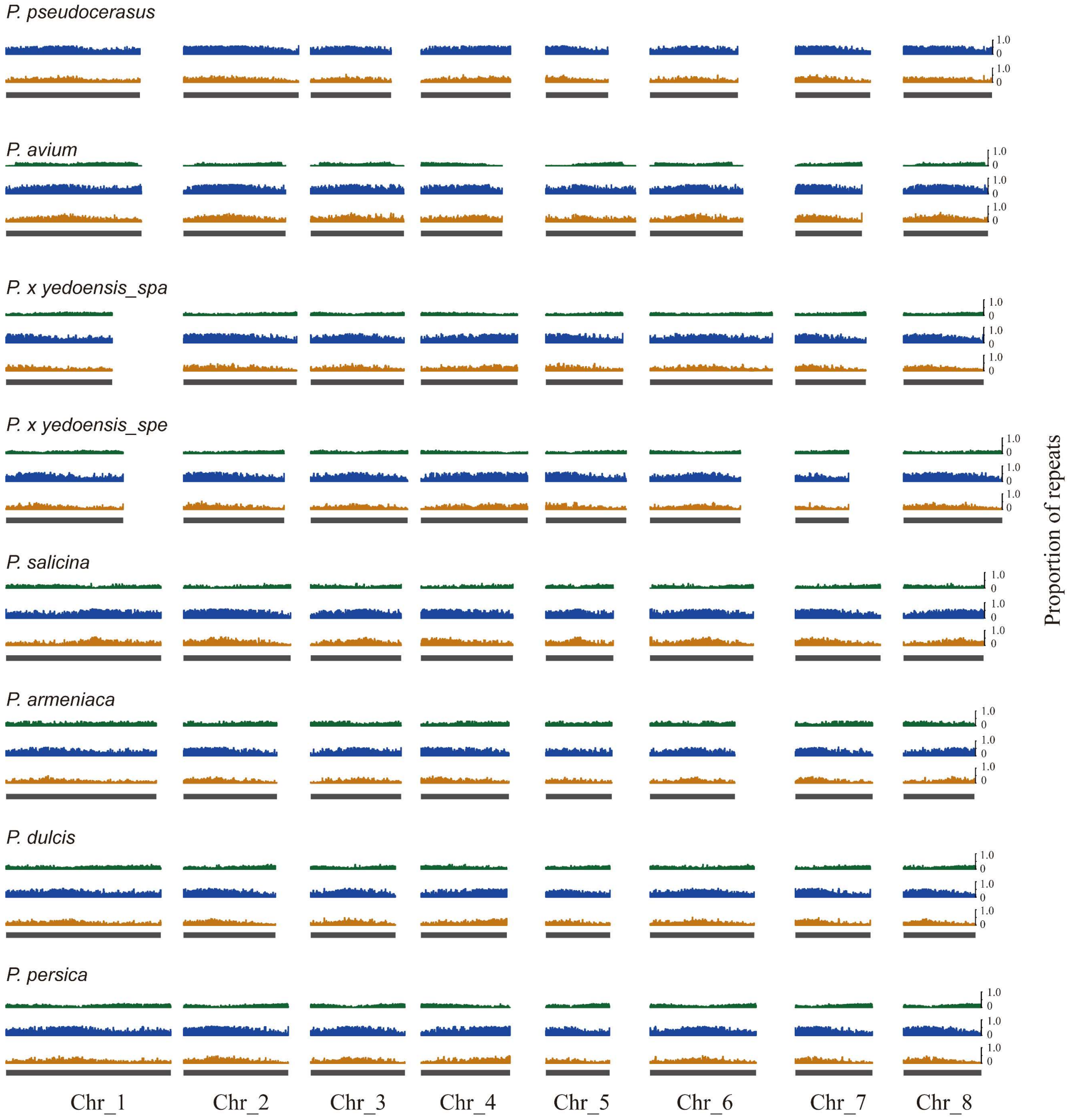

Supplementary Figure S1. Distribution of genes (track with green bar), TEs (track with blue bar) and LTRs (track with brown bar) on the pseudochromosomes of Chinese cherry and related species.

Note:

The tracks depict the content for different types sequences were estimated for 100kb windows.

An uncompleted gene annotation of Chinese cherry, and was not showed here.

Chromosome renamed for *P. salicina* and *P. armeniaca* follows the draft genome of peach.

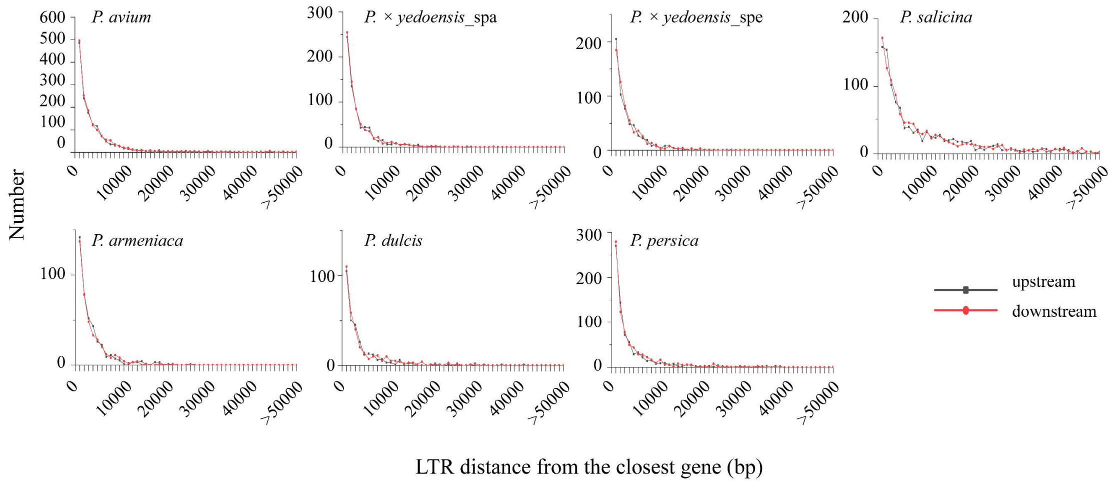

Supplementary Figure S2. Distribution of the distance between full-length LTR-RTs and genes across the species.

Note: Intergenic insertions are not counted. The grey line chart and red line chart indicated the upstream and downstream gene, respectively.

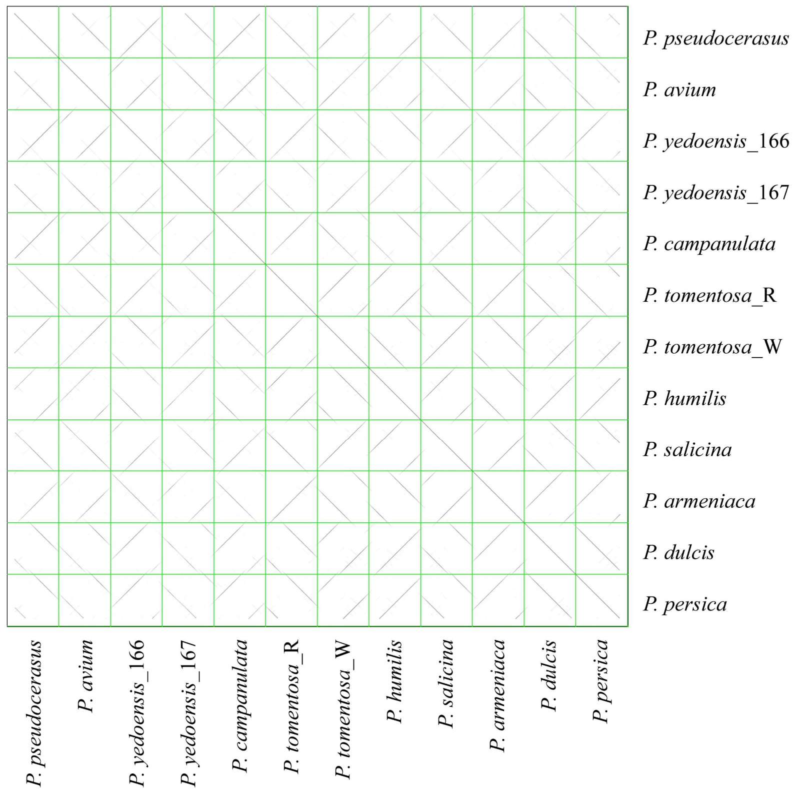

Supplementary Figure S3. Dot plots of the identified satellite repeat sequences with monomer units of 166 bp.

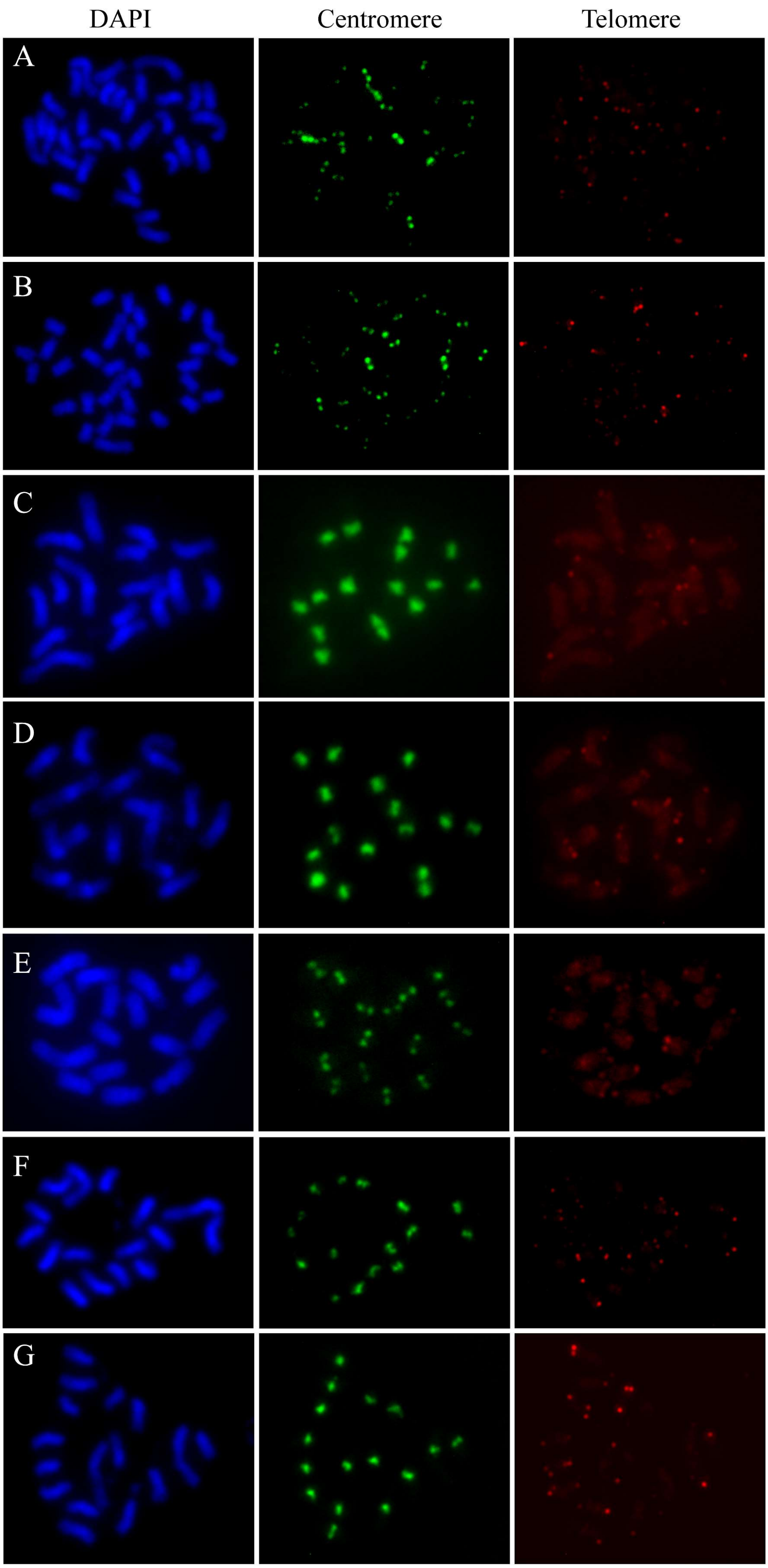

DAPI
Centromere
Telomere
A
B
C
D
E
F
G

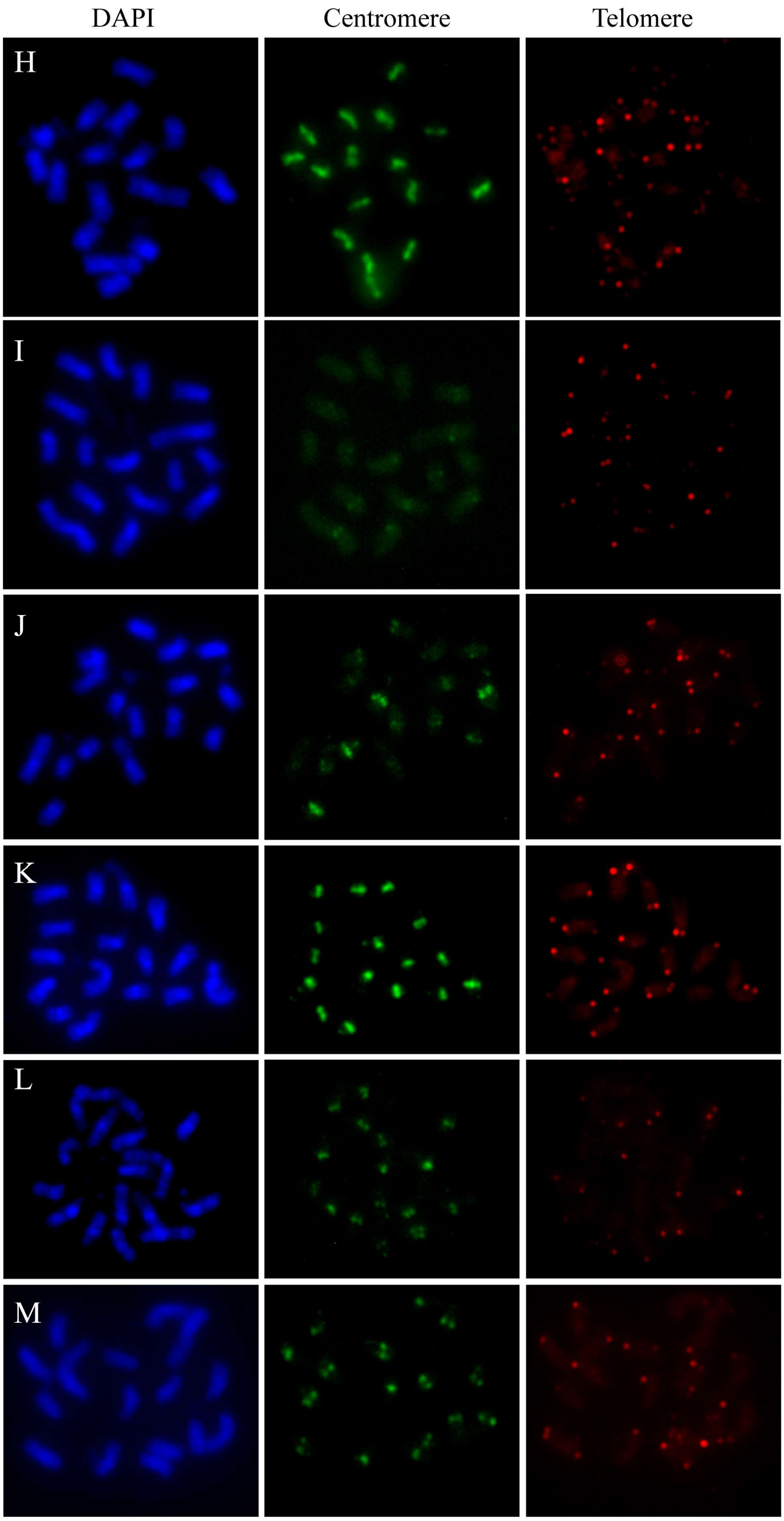
DAPI
Centromere
Telomere
H
I
J
K
L
M

Supplementary Figure S4. Chromosomal distribution and concentration comparison of oligonucleotide dye in 13 accessions from ten *Prunus* subgenus *Cerasus* species and related taxa.

Note: Green and red signals represented the distributions of centromeres and telomeres, respectively. A, B: *P. pseudocerasus,* HC and XC1; C, D: *P. avium*, 'Mazzard' and 'Van'; E: *P. campanulate*, Pcampan; F: *P. yedoensis*, Pyedoensis; G: *P. humilis*_Phumilis; H,I: *P. tomentosa*, red and white fruit; J: *P. salicina*, Cuihongli; K: *P. armeniaca,* diaogan; L: *P. dulcis*, Pdulcis; M: *P. persica*, Ppersica.

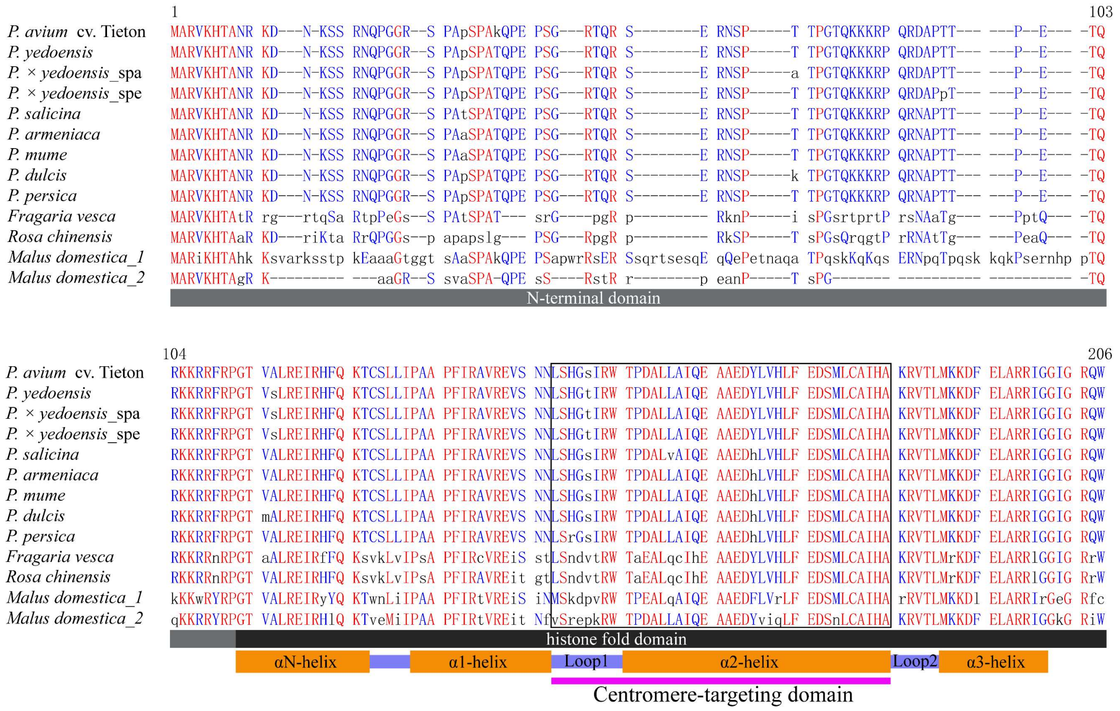

Supplementary Figure S5. Alignment of the protein sequences of CENH3 from eight *Prunus* subgenus *Cerasus* and relatives.

Note: Gray band below the alignment represented the N-terminal domain of the histone. Black band below the alignment stood for the histone fold domain. The secondary structure of the histone fold domain of the CENH3 was also exhibited below the alignment.
